# Chewing Affects Structural and Material Coupling, and Age-Related Dentoalveolar Joint Biomechanics and Strain

**DOI:** 10.3390/bioengineering13010093

**Published:** 2026-01-14

**Authors:** Haochen Ci, Xianling Zheng, Bo Wang, Sunita P. Ho

**Affiliations:** 1 State Key Laboratory of Structural Analysis for Industrial Equipment, Department of Engineering Mechanics, Dalian University of Technology, Dalian 116023, China; xianlingzheng@mail.dlut.edu.cn (X.Z.); 2 International Research Center for Computational Mechanics, Dalian University of Technology, Dalian 116023, China; 3 Department of Preventive and Restorative Dental Sciences, School of Dentistry, University of California, San Francisco, CA 94143, USA; haochen.ci@ucsf.edu (H.C.)

**Keywords:** biomechanics, joint stiffness, form, function, material properties, teeth, mechanical strain, food hardness

## Abstract

Understanding how primary structural features and secondary material properties adapt to functional loads is essential to determining their effect on changes in joint biomechanics over time. The objective of this study was to map and correlate spatiotemporal changes in primary structural features, secondary material properties, and dentoalveolar joint (DAJ) stiffness with age in rats subjected to prolonged chewing of soft foods versus hard foods. To probe how loading history shapes the balance between the primary and secondary features, four-week-old rats were fed either a hard-food (HF, N = 25) or soft-food (SF, N = 25) diet for 4, 12, 16, and 20 weeks, and functional imaging of intact mandibular DAJs was performed at 8, 12, 16, 20, and 24 weeks. Across this time course, the primary structural determinants of joint function (periodontal ligament (PDL) space, contact area, and alveolar bone socket morphology) and secondary material and microstructural determinants (tissue-level stiffness encoded by bone and cementum volume fractions, pore architecture, and bone microarchitecture) were quantified. As the joints matured, bone and cementum volume fractions increased in both the HF and SF groups but along significantly different trajectories, and these changes correlated with a pronounced decrease in PDL-space from 12 to 16 weeks in both diets. With further aging, older HF rats maintained significantly wider PDL-spaces than SF rats. These evolving physical features were accompanied by an age-dependent significant increase in the contact ratio in the SF group. The DAJ stiffness was significantly greater in SF than HF animals at younger ages, indicating that food hardness-dependent remodeling alters the relative contribution of structural versus material factors to joint function across the life course. At the tissue level, volumetric strains, representing overall volume changes, and von Mises bone strains, representing shape changes, increased with age in HF and SF joints, with volumetric strain rising rapidly from 16 to 20 weeks and von Mises strain increasing sharply from 12 to 16 weeks. Bone in SF animals exhibited higher and more variable strain values than age-matched HF bone, and changes in joint space, degrees of freedom, contact area, and bone strain correlated with joint biomechanics, demonstrating that multiscale functional biomechanics, including bone strain in intact DAJs, are colocalized with anatomy-specific physical effectors. Together, these spatiotemporal shifts in primary (structure/form), and secondary features (material properties and microarchitecture) define divergent mechanobiological pathways for the DAJ and suggest that altered loading histories can bias joints toward early maladaptation and potential degeneration.

## 1. Introduction

Daily functions including chewing are enabled by the dental, oral, and craniofacial masticatory system [1,2]. Chewing loads guide the development and growth of tissues within the masticatory complex [1,3]. The dentoalveolar joint (DAJ) is integral to the function of the masticatory complex. The DAJ consists of a tooth, alveolar bone, and the intervening periodontal ligament (PDL), an innervated and vascularized tissue that suspends the tooth within the alveolar socket [2]. Chewing loads beyond the normal physiological range cause significant changes in the cementum (the outer mineralized layer of a tooth root adjacent to dentin) of the tooth and alveolar bone volumes of the DAJ [4]. These changes in tissue volumes alter the morphology of the tooth root and alveolar socket and can result in overall changes in tooth movement relative to the bony socket and thereby alter DAJ biomechanics [5,6]. The magnitudes, frequencies, and duration of chewing loads are key regulators of the dynamic responses of these periodontal tissues [7,8,9,10]. Since Julius Wolff, it has been recognized that mechanical loads guide biological processes, driving adaptive changes in tissue morphology and material properties to accommodate function demands, though these responses may become maladaptive under abnormal or sustained loading conditions [11]. However, understanding how primary structural features and secondary material properties adapt to functional loads and how these affect joint biomechanics over time remains challenging.

Mechanoadaptation in alveolar bone, cementum, and the PDL is often investigated using small-scale animal models, such as rodents [1,12,13,14,15]. Studies leveraging the load-mediated structural response have mapped changes in bone mass, bone architecture, and PDL orientation and tissue turnover rates [14,16,17]. Furthermore, several studies have explored changes in structural features and/or material properties alongside biomechanical shifts in associated periodontal tissues [17,18]. By mechanically loading DAJs in situ and visualizing them using micro-X-ray computed tomography (micro-XCT), the physical interaction of tooth and alveolar socket under different mechanical stimuli has been characterized [19,20]. These functional imaging technologies have provided insights into tissue mechanics and morphology-mediated DAJ biomechanics. However, it remains poorly understood how primary structural features and secondary material properties adapt to functional loads and, in turn, shape joint biomechanics across age. The objective of this study is to map and correlate the spatiotemporal evolution of primary structural features, secondary material properties, and DAJ stiffness with age in rats subjected to prolonged chewing of soft foods and contrast with those fed hard foods. The results show that food-hardness-dependent effects on periodontal tissues reshapes primary and secondary features of DAJ function as animals age.

## 2. Materials and Methods

The University of California San Francisco (UCSF) Institutional Animal Care and Use Committee approved the experimental procedures used in this study (approval no. IACUC protocol AN200023-00I).

### 2.1. Animal Model and Experimental Workflow

**Figure 1:**
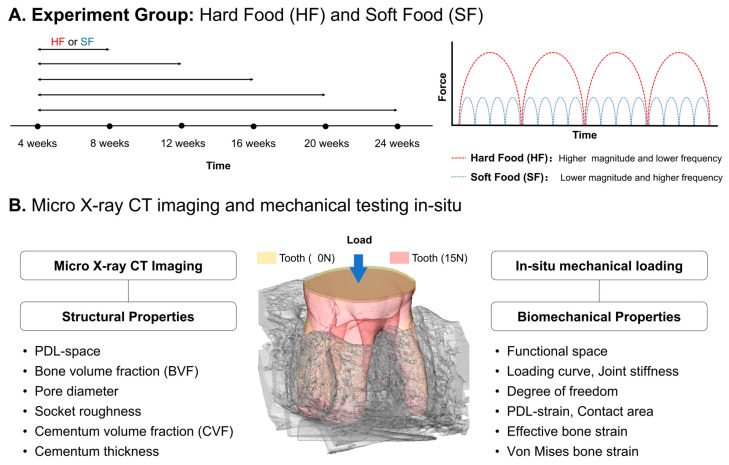
**Experimental workflow to assess structural and biomechanical properties of dentoalveolar joints (DAJs) using functional imaging.** (**A**) Experimental groups: rats were fed hard food (HF) or soft food (SF) and grown to 8, 12, 16, 20, and 24 weeks. Food hardness provided different magnitudes and frequencies of occlusal force. (**B**) Functional imaging assessed the physical properties of intact DAJs. Micro X-ray CT imaging characterized periodontal tissue structures, including PDL space, bone volume fraction (BVF), pore diameter (PD), socket roughness (SR), cementum volume fraction (CVF), and cementum thickness (CTh). Mechanical testing under load identified functional space, joint stiffness, degrees of freedom (DOF), PDL strain, contact area, and bone strain.

All experimental protocols were compliant with and followed the guidelines of the Institutional Animal Care and Use Committee (IACUC). The experimental workflow is shown in Figure 1. Sprague–Dawley male rats (N = 50; Charles River Laboratories, Inc., Wilmington, MA, USA) at 4 weeks of age were divided into 2 groups and fed with two nutritionally equivalent foods (PicoLab 5058, LabDiet, Deans Animal Feeds, Redwood City, CA, USA), which differed only in terms of hardness, hard pellet food (hard food (HF); N = 25) or soft powder chow (soft food (SF); N = 25). To measure the effects of food-hardness-mediated changes with age, rats were euthanized at 8, 12, 16, 20, and 24 weeks (Figure 1A). Right hemimandibles were isolated at each time point and were prepped to map DAJ stiffness and related periodontal tissue physical properties with age [4].

### 2.2. Biomechanical Testing In Situ

The harvested hemimandibles were prepared for biomechanical testing in situ. Compression tests of the prepared DAJ specimens were conducted in situ using the Deben loading device (MT500CT, Deben UK Ltd., Suffolk, UK). A TBS-soaked Kimwipe was placed around the DAJ specimen to ensure hydration during the test. In the compression test, each specimen was loaded to peak load (15 N) at a loading rate of 0.2 mm/min 4 times, and only the last 3 cycles were used. The outputs of force and displacement were processed to evaluate the biomechanical response of the DAJ. DAJ stiffness (N/mm) was calculated by approximating the final 30% of the load–displacement curve with a linear regression model [19].

### 2.3. Functional Imaging

After the compression test, another loading cycle was performed on each DAJ specimen coupled with a micro-X-ray computed tomography system (Micro XCT-200, Carl Zeiss X-ray Microscopy, Pleasanton, CA, USA). X-ray CT imaging of the DAJ specimens at no-load and peak-load stages were successively performed at 90 kVp, 7.8 W, 1200 projections, 4× magnification and 2 binning using a quartz silica (SiO_2_) filter. Tomographic images (16-bit grayscale image sequence) were reconstructed (XMReconstructor v8.1.6599, Xradia Inc., Pleasanton, CA, USA) to generate 3D volumes of the second molar and its periodontal complex at conditions of both no load and under load [19].

### 2.4. Structural Analysis via Image Processing

The tomographic images of periodontal tissues around the second mandibular molar were processed using AVIZO software (AVIZO 2019.4, FEI Visualization Sciences Group, Burlington, MA, USA) [21,22]. Cementum and bone pore volumes were segmented. The surfaces of the tooth and alveolar socket were generated using “generate surface” function. Structural parameters including bone volume fraction (BVF) (for tissue adaptation), pore diameter (PD) and tissue permeability, socket roughness (SR), cementum volume fraction (CVF), and cementum thickness (CTh) were estimated (Figure 1B) from the CT digital volumes [23]. We selected 100 μms into the alveolar bone at the mesial, distal, and interradicular regions (MR, DR, and IR) from the socket surface as the microanatomical region of interest (ROI). The structural parameters from coronal to apical regions of the mesial, distal, and interradicular regions (spatial: MR, DR, and IR), and across age (temporal: 8, 12, 16, 20 and 24 weeks) were normalized (scale of 0 (coronal) to 1 (apical)) and the trends with age were mapped.

### 2.5. PDL Strain and Contact Area Within a Loaded Periodontal Complex

The tether vectors at no-load (**T_0_**, 0 N) and load (**T_1_**, 15 N) were generated to represent the PDL-space and the functional space. X-ray tomograms from before and after loading were spatially registered to calculate the shifts in tethers (**T_s_**) as (**T_1_**–**T_0_**). The shifts were decomposed into two components: parallel (**T_II_**) and perpendicular (**T_Ʇ_**) shifts. The PDL normal strain (compression or tension) was estimated as the length of **T_II_** divided by the length of the original tether (**T_0_**). Compression and tension strain profiles in the PDL were identified as negative and positive strain values, respectively. Contact areas were identified as the PDL strain stiffening regions with compressive strain greater than 30%, as this provided a linear output in the load–displacement curve [5].

### 2.6. Digital Volume Correlation (DVC) to Map Strains in Alveolar Bone

The reconstructed volumetric images at 0 N and 15 N were filtered (to reduce image noise), registered (to remove rigid-body motion and rotation), and masked (to remove the tooth and keep the alveolar bone) using an in-house digital volume correlation software [24,25,26,27,28]. The image size of the interrogated volume for bone strain mapping was selected as ~750 × 750 × 750 voxel^3^ (~3.0 × 3.0 × 3.0 mm^3^) around the second mandibular molars. Six Cauchy strain components, including three normal strain components (ε_xx_, ε_yy_, ε_zz_) and three shear strain components (γ_xy_, γ_yz_, γ_zx_) were estimated. The subvolume size was chosen as 35 × 35 × 35 voxel^3^ for displacement tracking. The strain window size was specified as 11 × 11 × 11 points for strain mapping. Finally, the full-field distribution of maximum and minimum principal strain followed by the volumetric strain and equivalent von Mises strain within the alveolar bone were evaluated. The volumetric strains in alveolar bone represent a change in overall volume and von Mises bone strains represent a change in shape within a loaded alveolar bone.

### 2.7. Permeability Simulations

Regions of interest from the segmented micro-CT 3D image were processed into non-overlapping 50 × 50 × 100 voxels subvolumes. Intrinsic permeability (unit = μ^2^) was computed using Porous Microstructure Analysis (PuMA) version 3.0. for each subvolume location [29,30,31]. Permeability was based on the permeability homogenization function using a Finite Element method, which approximates both the velocity and pressure fields with first-order elements. Velocity field data from permeability computation was rendered using ParaView version 5.13.2 [31].

### 2.8. Statistical Analysis Including Principal Component Analysis

The Student’s *t*-test (unpaired, two-tail) was applied to determine significant differences between PDL-space, BVF, pore diameter, SR, CVF, and joint stiffness in the mesial region (MR), distal region (DR), and interradicular region (IR) between HF and SF groups with age. The *p*-value < 0.05 was used to highlight statistically significant differences between all parameters across HF and SF groups. Principal component analysis (PCA) on all measured physical parameters across HF and SF groups was performed.

## 3. Results

### 3.1. PDL Space and Functional Space

**Figure 2:**
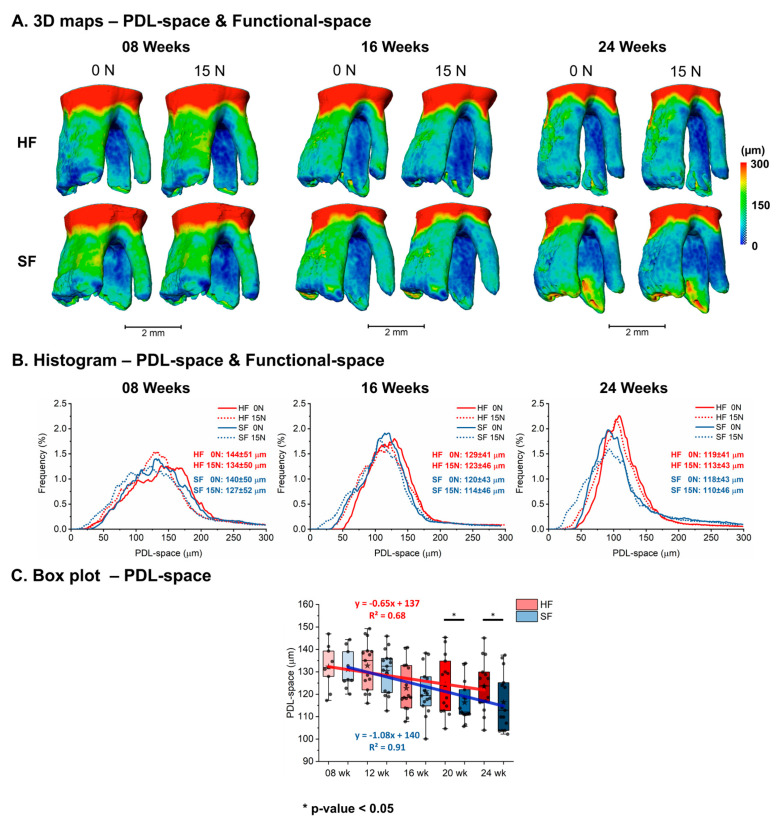
**Differential changes in joint space with age between HF and SF groups.** Joint space decreased with age and then stabilized in both groups; however, the SF group exhibited a narrower PDL-space, and a prolonged reduction period compared to the HF group. As expected, the mesial side (range from green to yellow) is wider than the narrower distal side (range from dark blue to green) (see scale bar from 0 mm dark blue to red 300 mm, to the right). (**A**) 3D maps of PDL-space at 0 N (unloaded) and functional-space at 15 N (loaded). (**B**) Histograms of PDL-space and functional-space distributions. (**C**) Average PDL-space in HF and SF groups across ages illustrate significant differences (* *p*-value < 0.05) between HF and SF PDL-space at 20 and 24 weeks of age.

Spatial maps of the PDL-space (no load–joint space) and functional-space (under load) revealed differences in the HF and SF groups (Figure 2A). PDL space showed overall decreasing trends in both groups. On average, functional space decreased but increased in variance with age in both the HF and SF groups (Figure 2B). However, a smaller average and wider range were observed in the SF group compared to the age-matched HF group (Figure 2C). A significant decrease in PDL-space from 12 to 16 weeks was observed in both HF and SF groups (HF: 133 ± 10 μm to 123 ± 10 μm, SF: 130 ± 9 μm to 120 ± 11 μm). However, a significant difference in PDL-space were observed between HF and SF groups at older ages (20 and 24 weeks) (Figure 2C).

### 3.2. Bone Volume Fraction, Pore Diameter, and Socket Roughness

Anatomy-specific differences in BVF in HF and SF groups were observed (Figure S1A). The BVF increased at younger ages and plateaued at older ages in HF but underwent a longer but slower increase in the SF (rate of increase between HF and SF is different). A significant increase in BVF from 8 to 12 weeks was observed in both HF and SF groups (HF: 76 ± 5% to 81 ± 6%, SF: 74 ± 7% to 79 ± 6%). Significant differences (*p*-value < 0.05) in BVF were found between HF and SF groups at 16 weeks (MR, DR, and IR) and 24 weeks (IR) (Figure S1B).

Spatial maps of pore skeleton and SR indicated anatomy-specific difference in alveolar bone structure in HF and SF (Figure S2A). The pore diameter and SR of the interradicular bone demonstrated a decreasing trend with age in both the HF and SF groups, and an increase in the SF group from 20 to 24 weeks (PD: 19 ± 5 μm to 24 ± 6 μm, SR: 17 ± 2 μm to 21 ± 3 μm) in all regions (MR, DR, and IR) (Figure S2B). Significant differences in pore diameter were observed between the HF and SF groups at 24 weeks in all regions (MR, DR, and IR) and 12 weeks only in the interradicular bone region (IR). The surface roughness between HF and SF was different at 24 weeks, with socket roughness from SF being higher than those from HF (MR, DR, and IR) (Figure S2B).

### 3.3. Permeability

To further characterize the interconnectivity of pores in the interradicular alveolar bone, longitudinal assessment of permeability was performed (Figure S3). In both HF and SF groups, bone permeability exhibited a progressive decline with aging, indicating a reduction in pore connectivity and fluid transport pathways. However, the HF and SF groups consistently showed higher permeabilities particularly at earlier time points, but SF suggested a delayed microstructural consolidation under reduced functional loads at 12 and 16 weeks. The 3D vector maps revealed a more heterogeneous and sparse distribution of permeable channels in the HF and SF with age (Figure S3A). Quantitative analysis confirmed significant temporal differences within each group, with a more gradual reduction in permeability observed in the SF group (* *p* < 0.05), reflecting the prolonged remodeling when chewing softer foods (Figure S3B).

### 3.4. Cementum Volume Fraction

Spatial maps of cementum volume revealed the anatomy-specific differences in CVF and cementum thickness from HF and SF (Figure S4A). The line profile of CVF from coronal to apical indicated a nonlinear distribution in MR, DR, and IR in both HF and SF groups at all five ages, indicating different growth rates of primary cementum compared to secondary cementum. Different growth rates of primary and secondary cementum are theorized to be affected by occlusal loads, and as indicated by the un-opposing tooth molar model (analogous to 0 N) [32,33]. CVF increased with age in both HF and SF groups, especially in mid and apical regions, and it was greater in the SF group than HF group, especially at older ages (Figure S3B). The histogram of cementum thickness illustrated an increase with age (HF: 58 ± 33 μm to 116 ± 64 μm, SF: 69 ± 41 μm to 125 ± 66 μm) and a larger average with a wider range in the SF group than in the HF group (Figure S4C). The boxplot of CVF suggested a global nonlinear increasing trend with age (HF: 20 ± 5% to 34 ± 4%, SF: 22 ± 2% to 38 ± 5%) but a higher rate of increase in the SF group than HF group. Significant differences in CVF were observed between the HF and SF groups at older ages (16, 20, and 24 weeks) (Figure S4D).

### 3.5. Joint Stiffness: Biomechanical Response of the DAJ 

**Figure 3:**
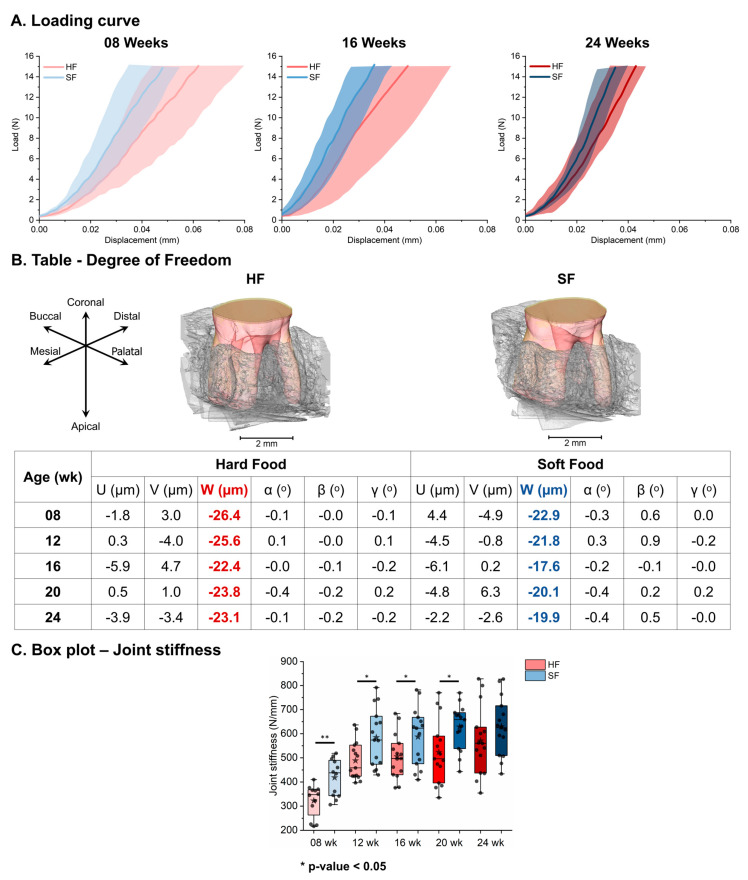
**Differences in joint stiffness and tooth movement between the HF and SF groups under functional loads.** The SF group exhibited higher rotational motion but lower displacement under functional loads, resulting in higher joint stiffness compared to age-matched HF groups. (**A**) Loading curves with 95% ± confidence intervals in HF and SF groups. (**B**) Six degrees of freedom of tooth movement within the alveolar socket under load. (**C**) Significant joint stiffness increase in SF compared to HF up to 20 weeks of age (* *p*-value < 0.05, ** *p* ≤ 0.01).

Load–displacement curves illustrated decreases in the displacement response and its variances with age in both HF and SF groups, and lower displacement responses in the SF group compared with the age-matched HF group (Figure 3A). A decreasing trend in tooth displacement with age was seen in both the HF and SF groups, but smaller tooth displacement and larger tooth rotation under load were detected in the SF group compared with the HF group (Figure 3B). The boxplot of joint stiffness was influenced by harder tissues and increased with age in both the HF and SF groups. However, a significantly higher joint stiffness (HF: 322 ± 64 N/mm to 570 ± 135 N/mm, SF: 418 ± 75 N/mm to 630 ± 115 N/mm) was observed in the SF group compared to the HF group up to 20 weeks (Figure 3C).

### 3.6. Contact Area

**Figure 4:**
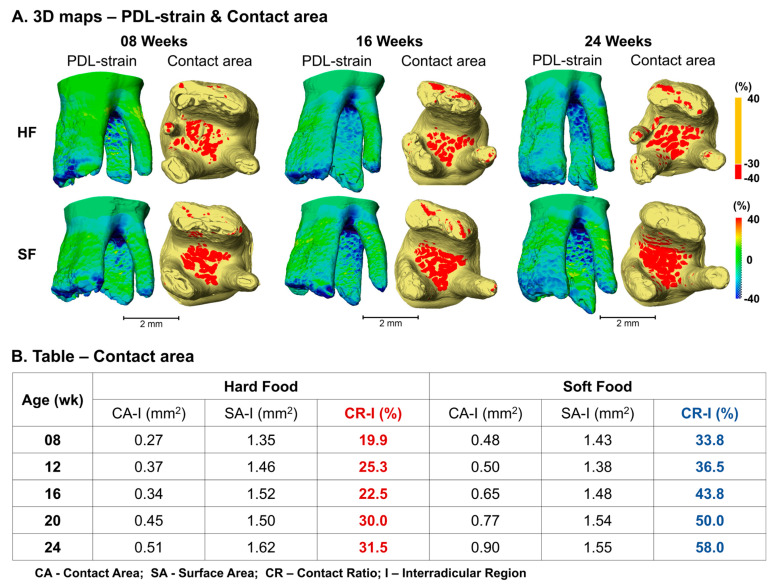
**Age-dependent changes in contact area and PDL strain between HF and SF groups.** Contact area increased with age in both groups; however, the SF group exhibited a larger contact area and a faster rate of increase compared to age-matched HF groups. (**A**) 3D maps of PDL strain and contact area. (**B**) Contact area and contact ratio in the interradicular (IR) region under load.

Spatial maps illustrated an increase in PDL strain and contact area with age, especially in IR for both HF and SF groups, and suggested larger stress transfer between the tooth and IR bone in the SF group compared with the age-matched HF group (Figure 4A). The contact ratio in IR listed in the table suggested an increasing trend with age in both HF and SF groups, and a larger increase at a higher rate with age in the SF group (33.8% to 58.0%) compared to the HF group (19.9% to 31.5%) (Figure 4B).

### 3.7. Bone Strain

**Figure 5:**
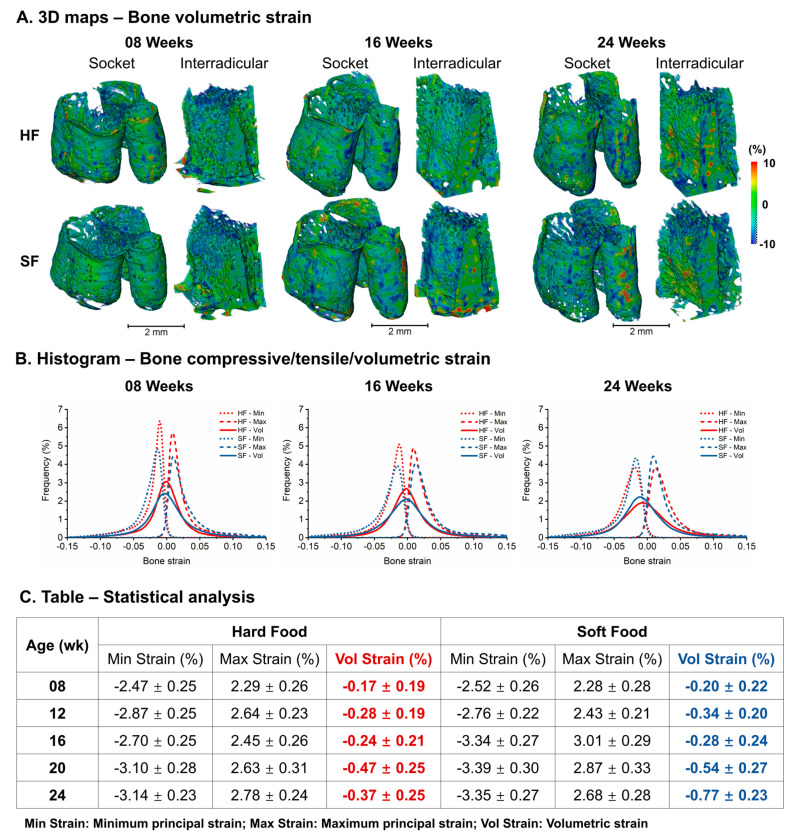
**Age-dependent changes in volumetric bone strain between HF and SF groups.** Volumetric bone strain increased with age, showing a rapid rise between 16 and 20 weeks; the SF group exhibited larger and more heterogeneous volumetric bone strain compared to age-matched HF groups. (**A**) 3D maps of bone volumetric strain in the alveolar socket and interradicular region. (**B**) Histograms of bone minimum principal, maximum principal, and volumetric strain in the HF and SF groups across ages. (**C**) Table showing mean ± SD of minimum principal, maximum principal, and volumetric strain.

Anatomy-specific differences in bone volumetric strain in both groups were observed. Larger bone volumetric strains were observed in the apical region in MR and DR, and the coronal region in IR at all five ages in both groups (Figure 5A). The histogram of bone strains suggested a larger average and wider range in the SF group compared to HF (Figure 5B). Bone volumetric strains illustrated an increasing trend with age and a rapid in-crease from 16 to 20 weeks in both HF and SF groups (HF: −0.24 ± 0.21% to −0.47 ± 0.25%, SF: −0.28 ± 0.24% to −0.54 ± 0.27%). The SF group indicated larger and more heterogeneous volumetric bone strain than the age-matched HF group (Figure 5C).

**Figure 6:**
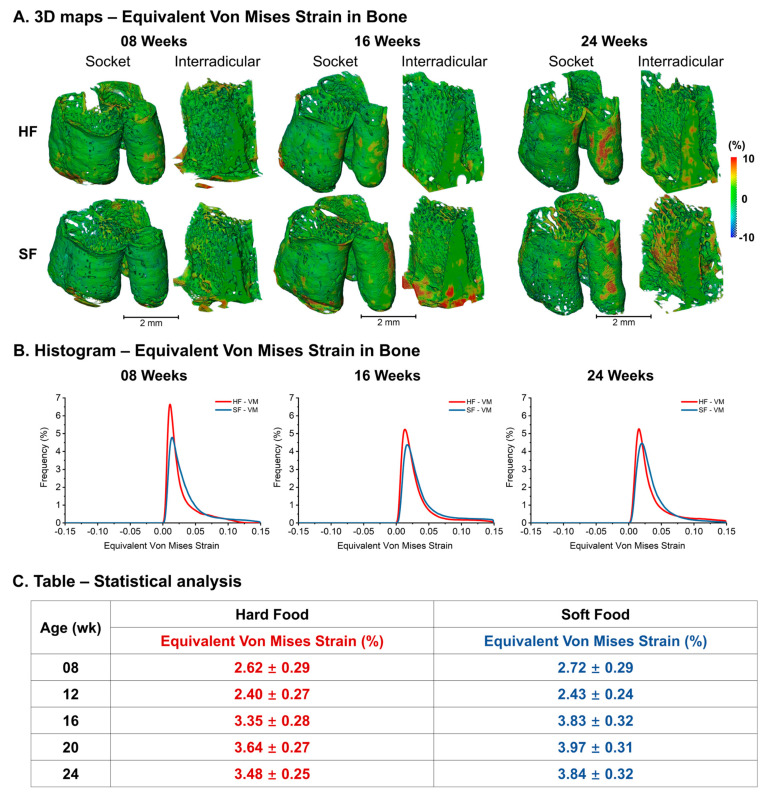
**Age-dependent changes in equivalent von Mises bone strain between HF and SF groups.** Equivalent von Mises bone strain increased with age, showing a rapid rise between 12 and 16 weeks; the SF group exhibited larger and more heterogeneous von Mises bone strain compared to age-matched HF groups. (**A**) 3D maps of equivalent von Mises bone strain in the alveolar socket and interradicular region. (**B**) Histograms of equivalent von Mises bone strain in HF and SF groups across ages. (**C**) Table showing mean ± SD of equivalent von Mises bone strain.

Bone von Mises strain within the alveolar socket and IR illustrated anatomy-specific differences in alveolar bone biomechanics (Figure 6A). A larger average and wider range in the SF group than in the HF group were observed (Figure 6B). Von Mises bone strains suggested an increasing trend with age and a rapid increase from 12 to 16 weeks in both the HF and SF groups (HF: 2.42 ± 0.27% to 3.35 ± 0.28%, SF: 2.43 ± 0.24% to 3.83 ± 0.32%). The SF group illustrated larger and more heterogeneous von Mises bone strain than the age-matched HF group (Figure 6C).

### 3.8. Correlation Analysis

**Figure 7:**
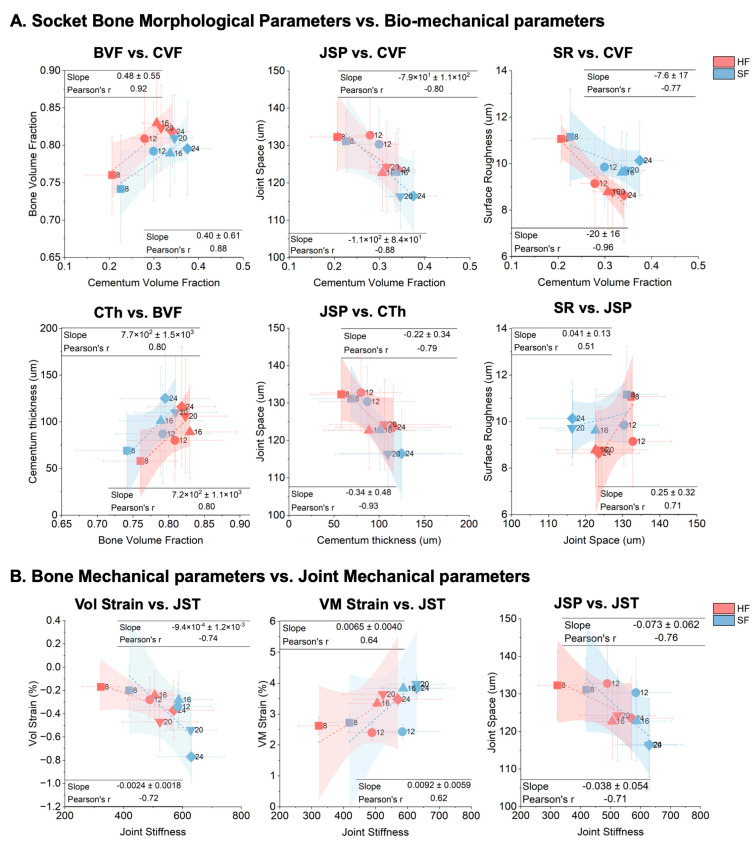
**Correlation analysis between morphological and biomechanical parameters of the dentoalveolar joint (DAJ).** (**A**) Correlations between socket morphological parameters and joint biomechanical parameters. (**B**) Correlations between bone and joint mechanical parameters. Each scatter plot represents SD values from individual specimens, color-coded by feeding group and age. Shaded regions indicate the 95% confidence interval of the linear regression. Regression slope and Pearson’s correlation coefficient (r) are reported in each panel.

Linear regression analyses revealed an age-related coordinated adaptation between bone socket morphology and the biomechanical properties of the tooth–bone complex, with distinct group-dependent differences. In both groups, BVF showed a strong positive correlation with CVF (HF: r = 0.92, SF: r = 0.88), but the HF group consistently exhibited higher BVF values than the SF group (Figure 7A). Joint space was negatively correlated with CVF (HF: r = −0.80, SF: r = −0.88) (Figure 7A). Similarly, socket bone surface roughness decreased with increasing CVF (HF: r = −0.96, SF: r = −0.77) (Figure 7A), implying smoother socket surfaces in HF due to sustained and higher magnitude mechanical stimulation compared to lower magnitude SF. Cementum thickness similar to CVF also was positively correlated with BVF (HF: r = 0.80, SF: r = 0.80) (Figure 7A), and joint space inversely correlated with cementum thickness (HF: r = −0.79, SF: r = −0.93) (Figure 7A). Moreover, bone surface roughness showed a moderate positive relationship with joint space (HF: r = 0.71, SF: r = 0.51) (Figure 7A), reflecting that SF samples, characterized by narrower joint gaps, retained rougher socket surfaces. Overall, the HF group demonstrated a socket architecture with larger joint gaps and smoother surfaces that contributed to lower joint stiffness, whereas the SF group exhibited narrower joint gaps with rougher surfaces that contributed to higher joint stiffness.

The volumetric strain (component of deformation associated with normal strain and volume change) showed significant negative correlations with joint stiffness (HF: r = −0.74, SF: r = −0.72) (Figure 7B) while von Mises strain (also known as effective strain, quantifies the shear-dominated part of the deformation) illustrated positive correlations (HF: r = 0.64, SF: r = 0.62) (Figure 7B). SF samples displayed higher stiffness and volumetric and von Mises strain magnitudes, suggesting that reduced masticatory loading results in deformable and yet questioning strength of tissues. In contrast, HF samples exhibited lower stiffness with reduced strain, indicating stiffer load-bearing behavior consistent with adaptive strengthening of mineralized tissues.

### 3.9. Principal Component Analysis

PCA was grouped by food hardness, integrating BVF, CVF, PD, and other structural parameters across all time points. (A) Score plots illustrate how HF and SF joints occupy distinct regions of the multivariate space. (B) The accompanying table summarizes the top three loadings contributing to PC1 and PC2 in the Overall, HF, and SF groups. For each group, variables with the largest absolute loadings were ranked to highlight the dominant factors shaping the primary (PC1) and secondary (PC2) variance structure. These rankings illustrate how the major sources of variation differ across groups and clarify the key variables driving the observed multivariate patterns.

**Figure 8:**
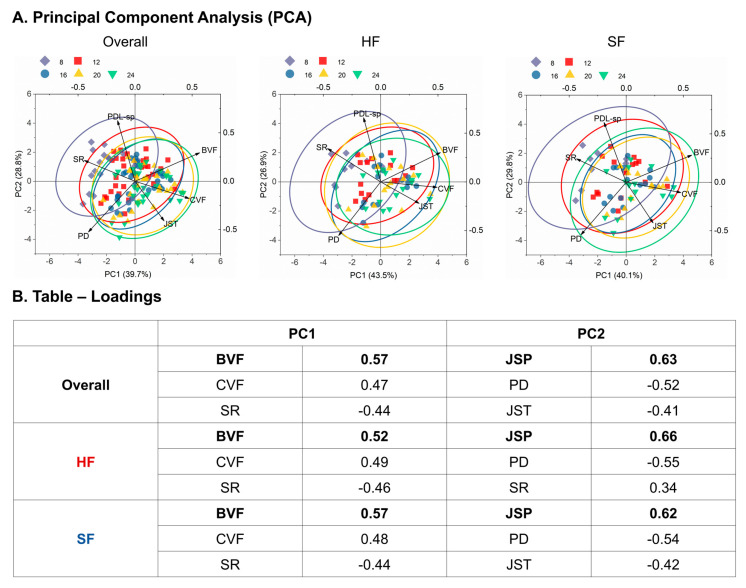
**Principal component analysis (PCA).** (**A**) PCA is grouped by food hardness, integrating bone volume fraction (BVF), cementum volume fraction (CVF), pore diameter (PD), and other structural parameters across all time points. (**B**) The table summarizes the top three loadings contributing to PC1 and PC2 in the Overall, HF, and SF groups. For each group, variables with the highest absolute loadings were ranked to highlight the dominant factors shaping the primary (PC1) and secondary (PC2) variance structure. These rankings illustrate how the major sources of variation differ across groups and clarify the key variables driving the observed multivariate patterns.

An age-related pattern emerged from the PCA. At eight weeks, surface roughness, PDL-space, and pore diameter dominated both HF and SF, indicating that early variation is primarily governed by joint geometry and surface characteristics (Figure 8A). By 24 weeks, BVF, CVF, and joint stiffness became the dominant contributors, suggesting that, with increasing duration of functional loading (age), variance shifts from early structural descriptors toward mineralized tissue fractions (BVF, CVF) and joint-level mechanical response (JST). If age is interpreted as a proxy for loading duration, these results are consistent with the idea that prolonged function primarily affects bone and cementum volume fractions and joint stiffness over time.

## 4. Discussion

Chewing on hard and soft foods produces distinct mechanical stimuli on the alveolar joint (Figure 1A). As expected, the data illustrated that, over time, the structural and biomechanical properties of periodontal tissues are interactive and regulate functional adaptation to meet the functional demands on the DAJ. In this study, the SF model was analogous to decoupling the dynamic motion of the tooth in the bony socket with the force on the tooth observed in the HF group. However, under the same simulated loading conditions on HF and SF adapted joints, the effect of sustained SF was observed as increased contact ratio between the tooth and the bone followed by increased volumetric and von Misses strains compared to a normally loaded tooth (hard pellets are the normal diet for rats, and softer foods are aberrant chewing loads).

During mastication, there is a force transfer path between periodontal tissues. The main load-transferring and load-bearing tissues involved in alveolar joints are cementum, PDL, and alveolar bone. Compressive loads push the tooth toward the alveolar socket, the shape and size of which will affect the degrees of freedom of the tooth [20,34]. Continuous pushing of the tooth following tooth–bone engagement causes PDL deformation [9]. The significant deformation (IR and apical regions are consistently observed to be the contact areas and sites of mechanoadaptation [26,34,35,36]) and mechanical strains in local areas encourages at first PDL strain stiffening followed by a high-level stress transfer to the alveolar bone. In this study, we mapped whether and how the load transfer pathway is affected by mapping changes in shape and tissue biomechanics as a function of two key and independent factors, (1) age (8, 12, 16, 20, and 24 weeks) and (2) food hardness (hard and soft foods).

### 4.1. Anatomy-Specific Morphology and Biomechanics Underlying Strain Transfer in the Periodontal Complex

The positive and negative associations between JSP, CVF, and bony SR indicate their net effect, which could influence PDL strain under loaded conditions at any given age. The PDL is strained as the tooth moves through the joint space in all three directions relative to the alveolar socket surface. In the apical MR/DR and coronal IR, the JSPs are relatively narrow, and, following loading, the functional spaces are significantly narrowed after the tooth moves towards the alveolar socket under compression, resulting in localized strain stiffening areas of PDL compression and stretched PDL. Although the PDL properties were not considered, it should be noted that PDL turnover rates resulting from reduced chewing loads could affect its mechanical integrity and influence the contact area. Experimental results suggested an increasing trend of compressive PDL strain with age in both HF and SF groups (Figure 4A,B). Compared with the HF group, the joint space of the SF group was narrower (Figure 2B,C) with relatively fewer degrees of freedom, as indicated by shorter tooth movements within the alveolar socket (Figure 3B). Under their collaborative influence (joint space and degrees of freedom), the PDL strain in the SF group was larger on average, and this shift was more significant in the coronal IR, thus yielding larger contact areas compared with the age-matched HF group (Figure 4B).

The observed bone strain because of the contact is mainly governed by the distribution of bone structure (represented by BVF), tissue property contact stress (represented by joint stiffness, JST) [23,34,36]. The BVF underwent two temporal evolution stages: growth stage (younger age: 8 to 12 weeks) and stable stage (older age: 12–24 weeks) (Figure 7A and Figure S1B). The contact area demonstrated an increasing trend with age (Figure 4B), suggesting significantly increased contact stress between the tooth and alveolar bone in SF group. Under their collaborative influences, an increasing trend of volumetric strain (compressive) and equivalent von Mises strain (shear) with age were observed (Figure 5C and Figure 6C). Regardless, a moderate change in deformation resistance (volumetric strain) for a significant increase in joint stiffness with age for HF compared to a steep change in deformation resistance for a small increase in joint stiffness for SF (Figure 7B) indicates the mechanical vulnerability of the SF compared to a resilient condition of the HF group.

### 4.2. Association Between Tissue-Level Transformation and Joint-Level Function

Under the integrative adaptation of BVF and CVF, narrower joint space and hence higher joint stiffness were observed in the SF group compared with the age-matched HF group. It is worth noting that, at younger ages, the joint stiffness in HF and SF groups was significantly different (Figure 3C), while the related joint spaces and periodontal tissue structures (BVF, pore diameter, and CVF) (Figures S1B, S2B and S4D) did not show significant differences, which might be attributed to tissue adaptation. In contrast, at older ages, the difference in joint stiffness between HF and SF groups weakened (Figure 3C), but the periodontal tissue structures showed significant differences between HF and SF groups (Figures S1B, S2B and S4D). Therefore, it is worth noting the shift in indicators with age, that is, from changes in joint stiffness at younger ages to changes in tissue morphology at older ages. The results demonstrated the dominance of material-induced differences at younger ages, and the governance of structure-induced differences at older ages in the SF group when compared to the HF group.

### 4.3. Functional Load, Strain, and Structure–Material Pathways

In both HF and SF, the functional load condition did not directly alter which variables were classified as primary versus secondary factors, but it did highlight that shear (von Mises) and volumetric strains can play a dominant role in modifying surface roughness, BVF, and CVF. For HF, the inferred mechanobiological pathway emphasizes the evolution of joint space and surface roughness, in relation to pore diameter, BVF, and CVF. Along PC1, variables clustered by age (8–24 weeks). At younger ages, PDL space, surface roughness, and pore diameter were the dominant contributors, whereas, at older ages, BVF, CVF, and joint stiffness became predominant.

For SF, the pathway emphasizes the evolution of joint space and joint stiffness, again in relation to pore diameter, with downstream effects on surface roughness, BVF, and CVF. Thus, while both HF and SF share common variables, the relative weighting and temporal ordering of structural and material factors differ by diet.

### 4.4. Food-Hardness-Dependent Differences in Strain and Implied Material Behavior

PDL space, BVF, CVF, and volumetric and von Mises bone strains were all different in SF at older ages compared to HF. Notably, when changes in physical parameters (e.g., BVF and CVF) were similar between HF and SF, SF still exhibited higher strains. This suggests that differences in apparent material behavior may emerge despite CVF being higher in SF than HF.

These observations raise questions about the role of contact area and geometric constraint. Since the effective degrees of freedom and load transfer are partly encoded by contact area, the higher contact area in SF relative to HF provides a plausible explanation for the elevated strain state in SF joints. Together, these findings support the view that the biomechanics of SF DAJs, as related to both material properties and contact mechanics, are distinct from HF, with SF joints occupying a more mechanically vulnerable, high-strain regime at older ages.

## 5. Conclusions

Our data provided insights into an altered biomechanical pathway in the SF group compared to the HF group and alluded to mechanical vulnerability. Food-hardness-dependent effects on periodontal tissues reshape primary structural and secondary determinants as animals age. Prolonged soft-food chewing drives a trajectory characterized by increased joint stiffness and contact ratio, constrained PDL space, and amplified, heterogeneous bone strains, indicating a mechanically vulnerable state, whereas hard-food chewing supports a trajectory in which the enlargement of PDL space and more moderate changes in strain accompany maturation. Together, these HF and SF pathways illustrate how differences in chewing history bias the balance between structure, material properties, and contact mechanics, steering DAJs toward either a more resilient or a more mechanically vulnerable multiscale strain environment. As the central objective of this study was to investigate the effect of food hardness on DAJ biomechanics and correlate with potential changes in tissue properties, we limited our study to male rats. Subsequent studies including females, but which also map hormone associated changes in bone and related periodontal tissues, are warranted. Future histological assessments will help delineate between adaptive remodeling and early maladaptive or degenerative processes, particularly to aberrant loads, such as soft food, in this study. The integration of finite element modeling with digital volume correlation will further refine quantitative insights into its functional adaptation under altered mechanical environments.

## Data Availability

The raw data supporting the conclusions of this article will be made available by the authors on request. The data are not publicly available due to the volume of data, and as such requested specific data will be provided.

## Supplementary Materials

The following supporting information can be downloaded at: https://www.mdpi.com/article/10.3390/bioengineering13010093/s1, Figure S1. BVF increased with age and then remained stable in both HF and SF groups; the SF group showed lower BVF and a slower increasing trend than the HF group. (A) 3D maps of bone and pore volumes in the alveolar socket and interradicular region. (B) Average BVF in HF and SF groups with age. MR: mesial region, DR: distal region, IR: interradicular region. Figure S2. Pore diameter and socket roughness showed a global decrease trend with age; the SF group showed larger pore diameter and socket roughness than the HF group. (A) 3D maps of pore diameter and socket roughness. (B) global average pore diameter and socket roughness in HF and SF groups with age. MR: mesial region, DR: distal region, IR: interradicular region. Figure S3. Interradicular bone permeability decreased progressively with age in both HF and SF groups, with the HF at 12 weeks showing relatively higher permeability values than SF. Heterogeneous distribution of permeability at each time point in both HF and SF were observed. (A) 3D vector maps of interradicular bone permeability at 8, 16, and 24 weeks. (B) Box plots of permeability values in the interradicular region across different ages, indicating significant changes as 12 weeks (* *p* < 0.05). Figure S4. CVF increased with age in both HF and SF groups; the SF group showed higher CVF and a longer increase period and faster nonlinear rate of increase than the HF group. (A) 3D maps of cementum volume and cementum thickness. (B) CVF in MR, DR, and IR from coronal to apical. (C) Histogram of cementum thickness in HF and SF groups with age. At 8 weeks, SF illustrates an increased variance in cementum thickness compared to age-matched HF. (D) Average CVF in HF and SF groups with age. Note: Arrow indicates growth over time–red profile from 8 weeks to blue profile 24 weeks demonstrate a rapid increase in cementum thickness for a small increase in root length specifically in the distal and interradicular regions compared to mesial region in both HF and SF. Figure S5. Age-dependent variance in correlations between morphological and biomechanical parameters. (A) Temporal evolution of socket bone morphological parameters relative to biomechanical indices. (B) Age-related changes in bone mechanical parameters relative to joint mechanical responses. Each scatter represents mean ± SD from the hard-food (HF, red) and soft-food (SF, green) groups at 8, 12, 16, 20, and 24 weeks. Blue dashed lines denote the inter-group difference (Diff) between HF and SF trends.
