# Supplementary material for: Chewing Affects Structural and Material Coupling, and Age-Related Dentoalveolar Joint Biomechanics and Strain
Source: Bioengineering (Basel). 2026 Jan 14;13(1):93. doi: 10.3390/bioengineering13010093 (PMC12838062; doi:10.3390/bioengineering13010093)
Supplement: Supplementary file 1 [file bioengineering-13-00093-s001.zip › bioengineering-4041869-supplementary.pdf]

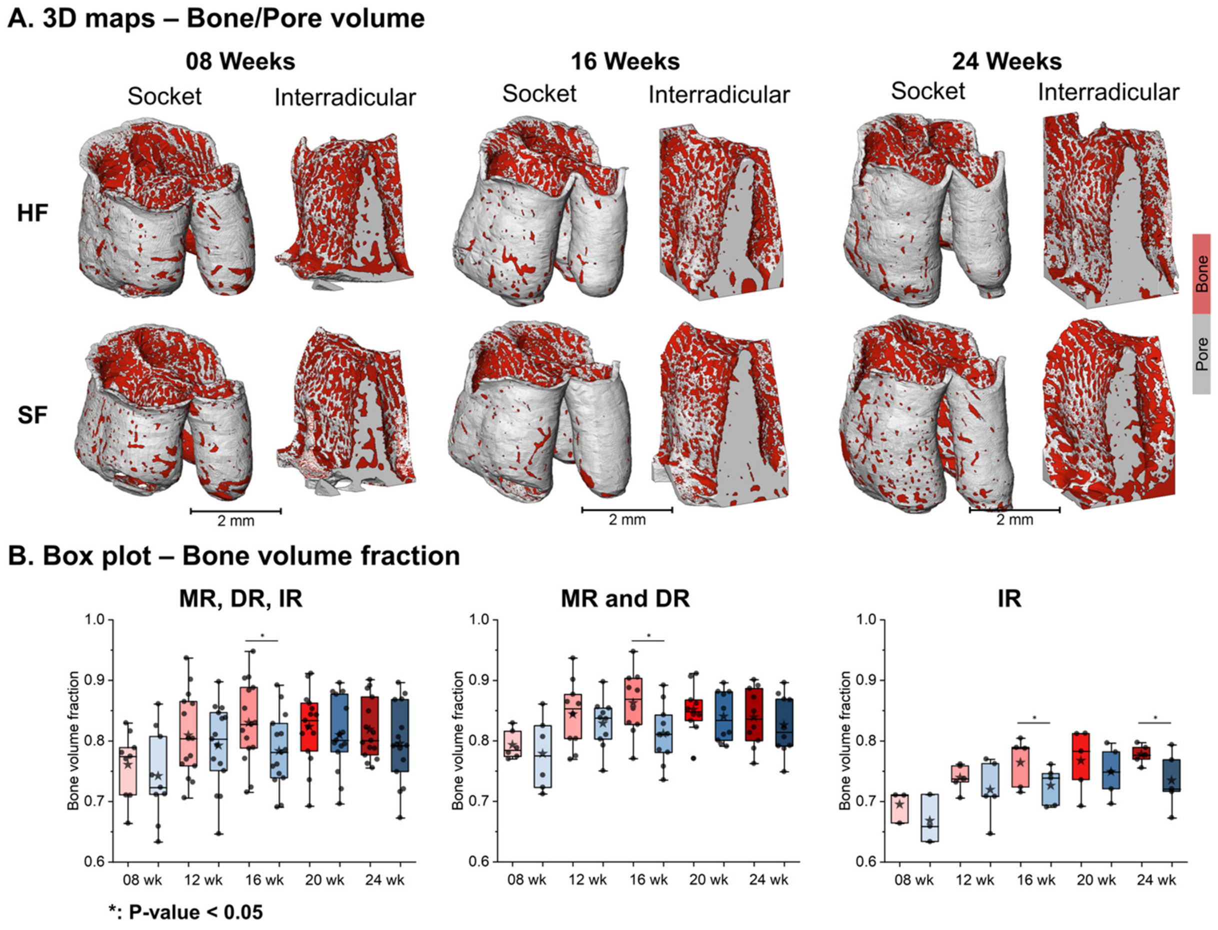

Figure S1. BVF increased with age and then remained stable in both HF and SF groups; the SF group showed lower BVF and a slower increasing trend than the HF group. **(A)** 3D maps of bone and pore volumes in the alveolar socket and interradicular region. **(B)** Average BVF in HF and SF groups with age. MR: mesial region, DR: distal region, IR: interradicular region.

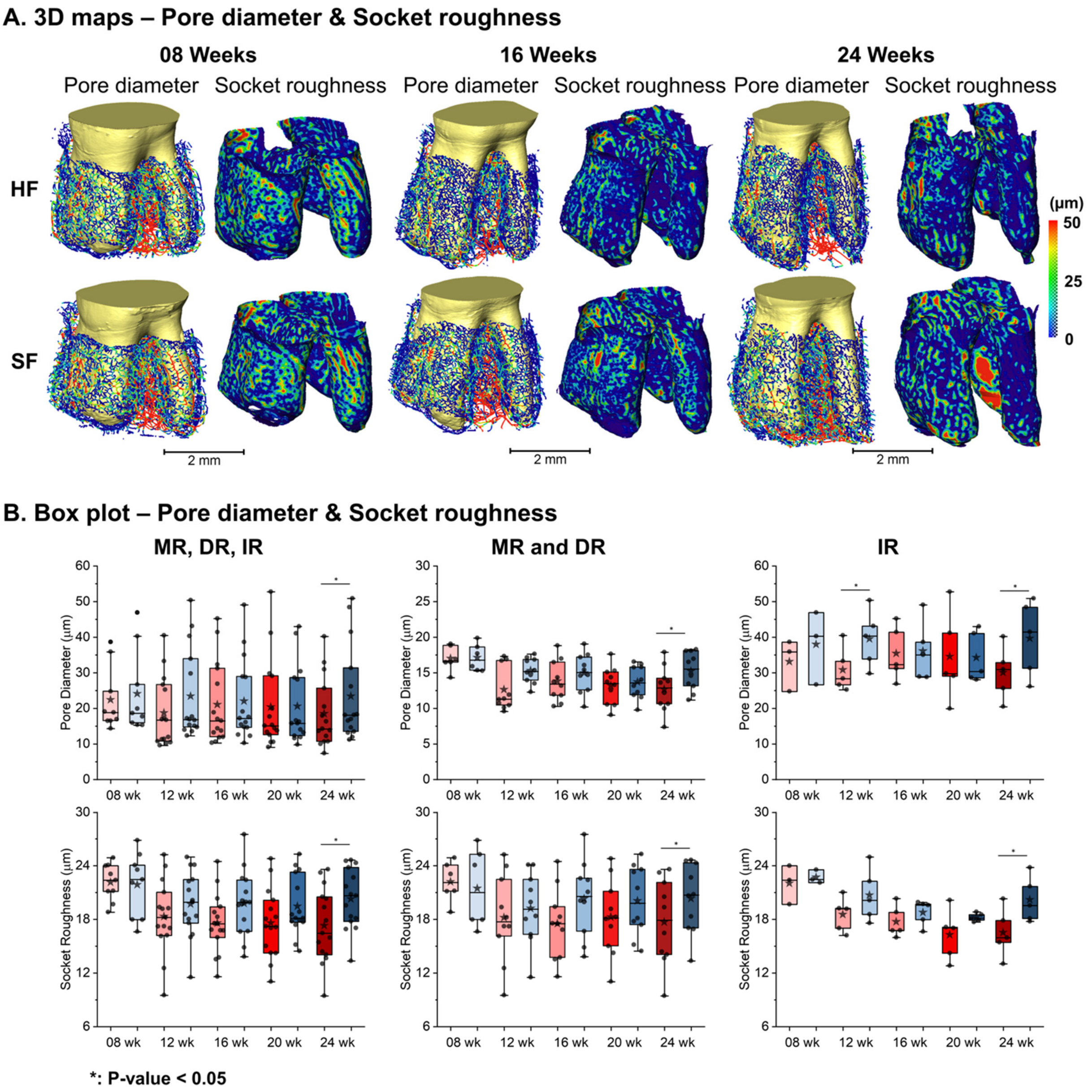

Figure S2. Pore diameter and socket roughness showed an overall decreasing trend with age; the SF group showed larger pore diameter and socket roughness than the HF group. **(A)** 3D maps of pore diameter and socket roughness. **(B)** Global average pore diameter and socket roughness in HF and SF groups with age. MR: mesial region, DR: distal region, IR: interradicular region. **(C)** Longitudinal and transverse sections of the periodontal complex illustrate physical features that contribute to the surface roughness at the mesial and distal sides of the periodontal complex.

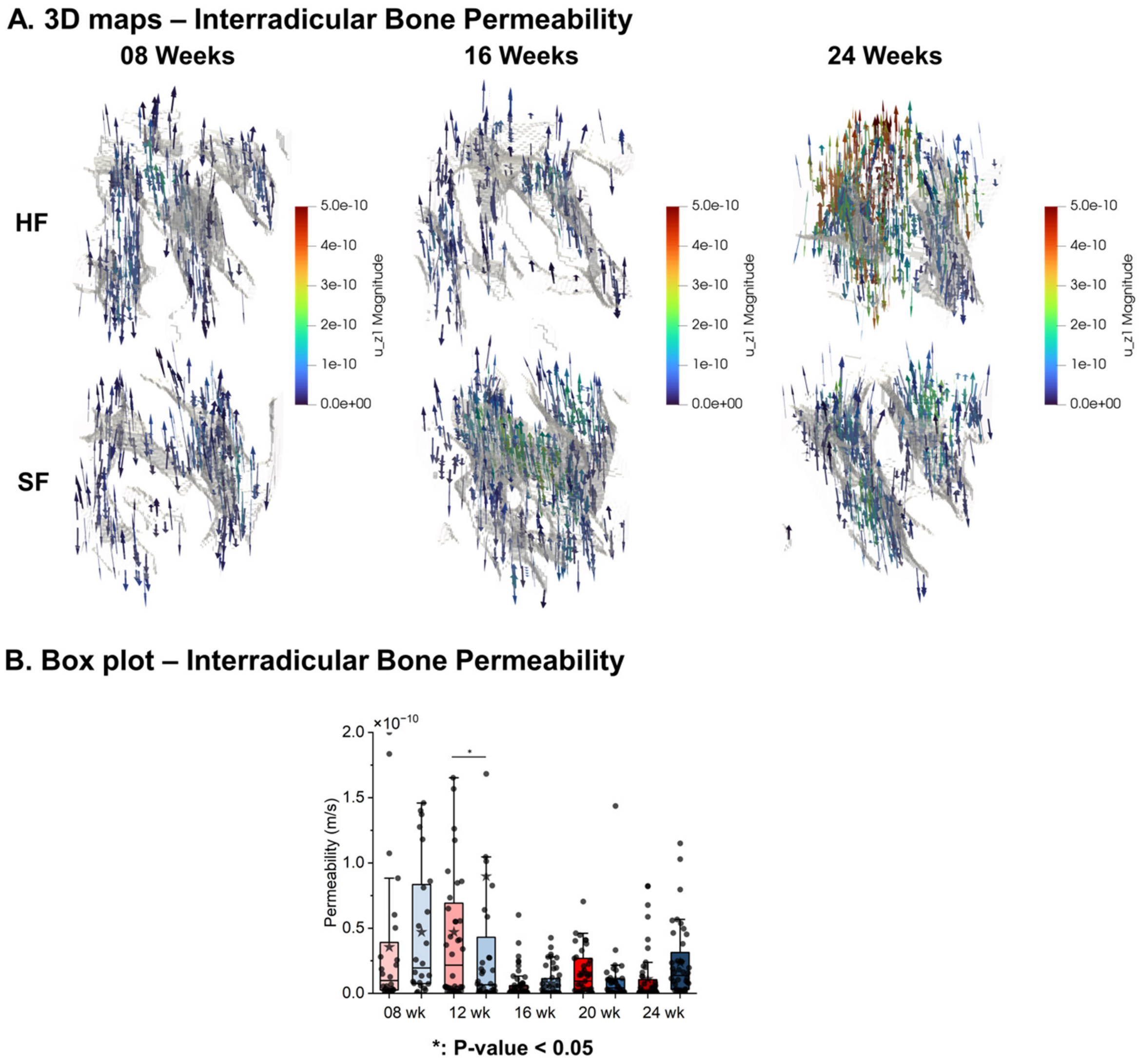

Figure S3. Interradicular bone permeability decreased progressively with age in both HF and SF groups, with the HF at 12 weeks showing relatively higher permeability values than SF. Heterogeneous distribution of permeability at each time point in both HF and SF were observed. **(A)** 3D vector maps of interradicular bone permeability at 8, 16, and 24 weeks. **(B)** Box plots of permeability values in the interradicular region across different ages, indicating significant changes as 12 weeks (*$P < 0.05$).

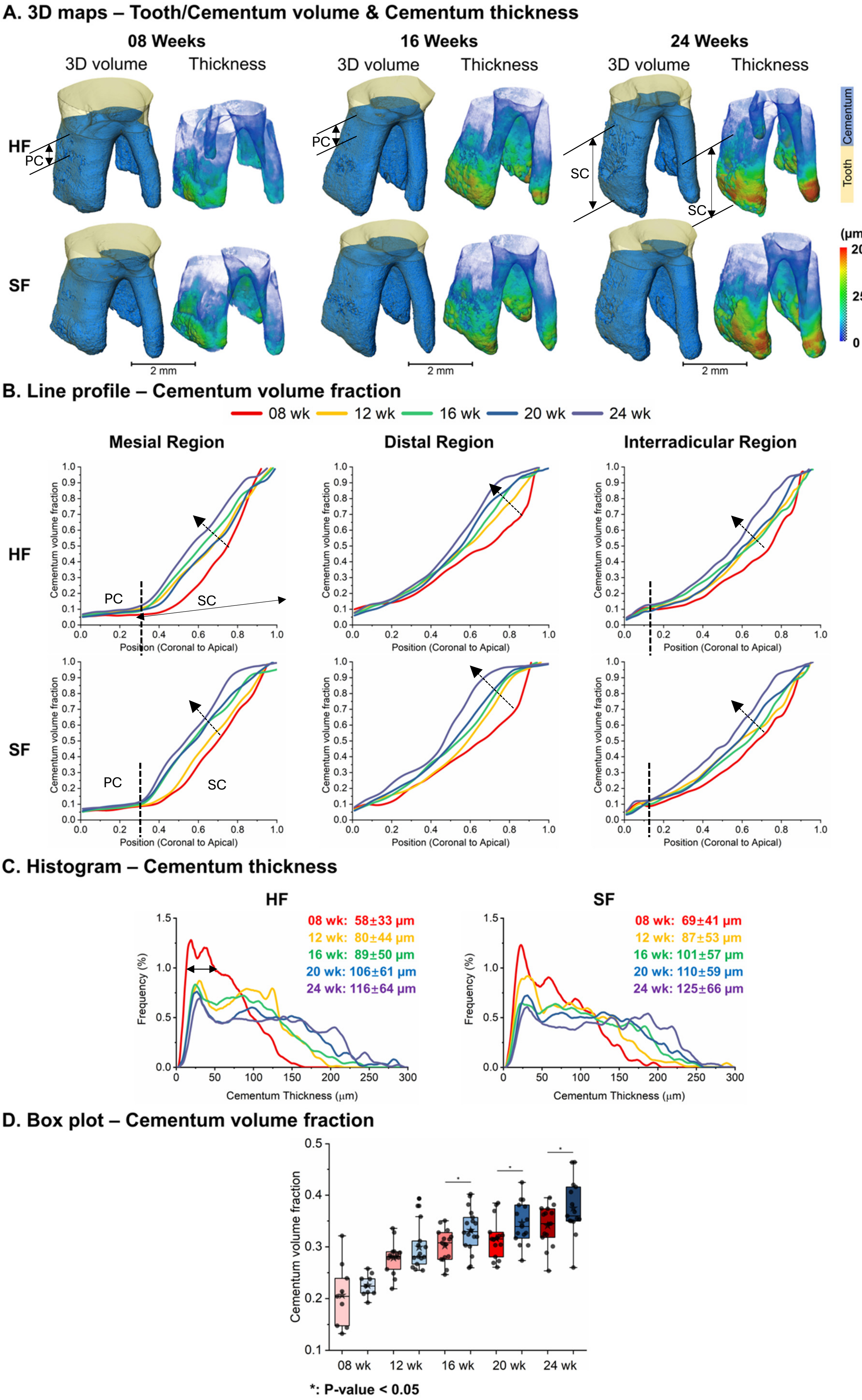
A. 3D maps – Tooth/Cementum volume & Cementum thickness
08 Weeks
16 Weeks
24 Weeks
3D volume
Thickness
HF
SF
PC
SC
Cementum
Tooth
(μm)
200
25
0
2 mm
B. Line profile – Cementum volume fraction
08 wk
12 wk
16 wk
20 wk
24 wk
Mesial Region
Distal Region
Interradicular Region
Cementum volume fraction
Position (Coronal to Apical)
C. Histogram – Cementum thickness
HF
08 wk: 58±33 μm
12 wk: 80±44 μm
16 wk: 89±50 μm
20 wk: 106±61 μm
24 wk: 116±64 μm
SF
08 wk: 69±41 μm
12 wk: 87±53 μm
16 wk: 101±57 μm
20 wk: 110±59 μm
24 wk: 125±66 μm
Frequency (%)
Cementum Thickness (μm)
D. Box plot – Cementum volume fraction
Cementum volume fraction
08 wk
12 wk
16 wk
20 wk
24 wk
*: P-value < 0.05

Figure S4. **CVF increased with age in both HF and SF groups**; the SF group showed higher CVF and a longer increase period and faster nonlinear rate of increase than the HF group. **(A)** 3D maps of cementum volume and cementum thickness. **(B)** CVF in MR, DR, and IR from coronal to apical. **(C)** Histogram of cementum thickness in HF and SF groups with age. At 8 weeks, SF illustrates an increased variance in cementum thickness compared to age-matched HF. **(D)** Average CVF in HF and SF groups with age. **Note:** Arrow indicates growth over time – red profile from 8 weeks to blue profile 24 weeks demonstrate a rapid increase in cementum thickness for a small increase in root length specifically in the distal and interradicular regions compared to mesial region in both HF and SF.

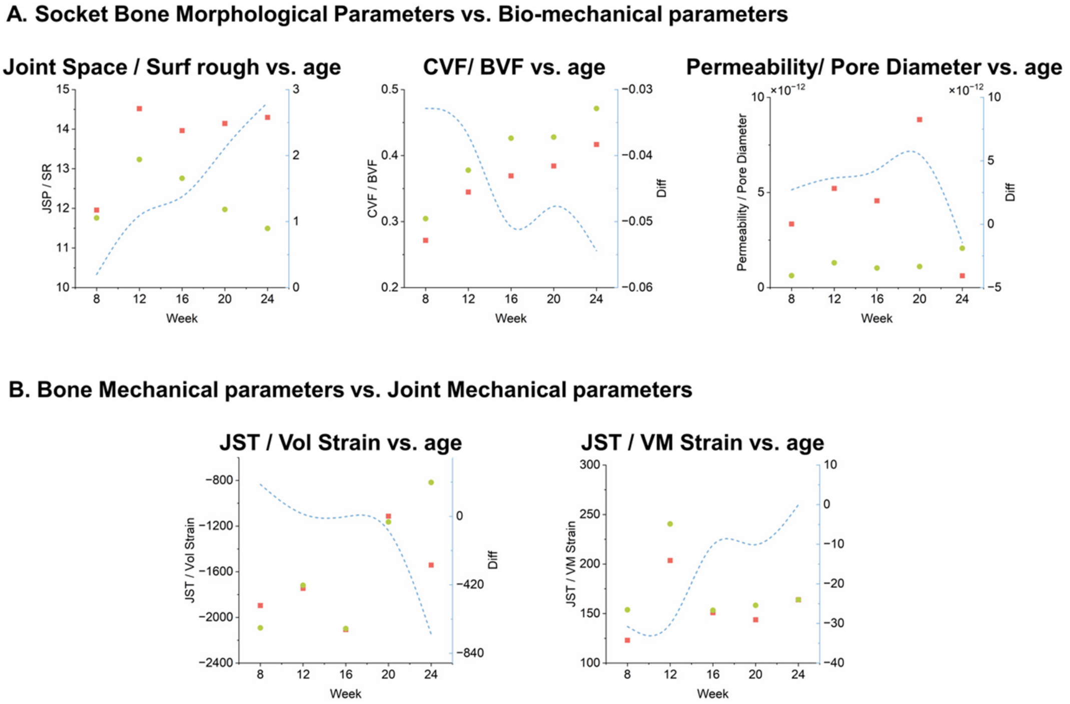

Figure S5. Age-dependent variance in correlations between morphological and biomechanical parameters. (A) Temporal evolution of socket bone morphological parameters relative to biomechanical indices. **(B)** Age-related changes in bone mechanical parameters relative to joint mechanical responses. Each scatter represents mean ± SD from the hard-food (HF, red) and soft-food (SF, green) groups at 8, 12, 16, 20, and 24 weeks. Blue dashed lines denote the inter-group difference (Diff) between HF and SF trends.
